# Role of the TWEAK-Fn14-cIAP1-NF-κB Signaling Axis in the Regulation of Myogenesis and Muscle Homeostasis

**DOI:** 10.3389/fimmu.2014.00034

**Published:** 2014-02-05

**Authors:** Emeka K. Enwere, Eric C. LaCasse, Nadine J. Adam, Robert G. Korneluk

**Affiliations:** ^1^Department of Medical Microbiology and Immunology, University of Alberta, Edmonton, AB, Canada; ^2^Solange Gauthier Karsh Molecular Genetics Laboratory, Apoptosis Research Centre, Children’s Hospital of Eastern Ontario Research Institute, Ottawa, ON, Canada; ^3^Department of Biochemistry, Microbiology and Immunology, University of Ottawa, Ottawa, ON, Canada

**Keywords:** TWEAK, Fn14, cIAP1, NF-κB signaling, myogenesis, myoblast fusion, muscle regeneration

## Abstract

Mammalian skeletal muscle maintains a robust regenerative capacity throughout life, largely due to the presence of a stem cell population known as “satellite cells” in the muscle milieu. In normal conditions, these cells remain quiescent; they are activated upon injury to become myoblasts, which proliferate extensively and eventually differentiate and fuse to form new multinucleated muscle fibers. Recent findings have identified some of the factors, including the cytokine TNFα-like weak inducer of apoptosis (TWEAK), which govern these cells’ decisions to proliferate, differentiate, or fuse. In this review, we will address the functions of TWEAK, its receptor Fn14, and the associated signal transduction molecule, the cellular inhibitor of apoptosis 1 (cIAP1), in the regulation of myogenesis. TWEAK signaling can activate the canonical NF-κB signaling pathway, which promotes myoblast proliferation and inhibits myogenesis. In addition, TWEAK activates the non-canonical NF-κB pathway, which, in contrast, promotes myogenesis by increasing myoblast fusion. Both pathways are regulated by cIAP1, which is an essential component of downstream signaling mediated by TWEAK and similar cytokines. This review will focus on the seemingly contradictory roles played by TWEAK during muscle regeneration, by highlighting the interplay between the two NF-κB pathways under physiological and pathological conditions. We will also discuss how myogenesis is negatively affected by chronic conditions, which affect homeostasis of the skeletal muscle environment.

## Introduction

**Figure 1:**
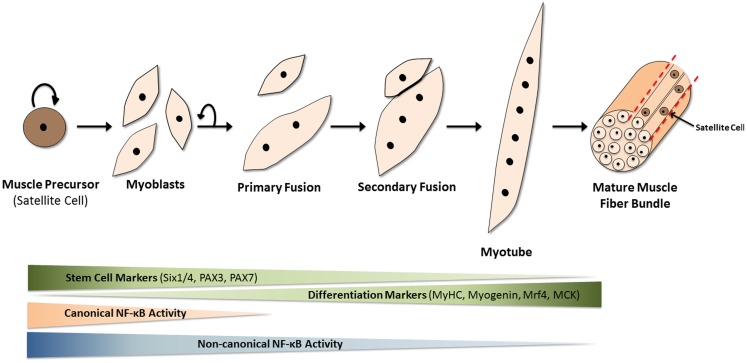
**Mammalian skeletal myogenesis**. Upon muscle injury, a resident population of quiescent myogenic precursor cells, known as satellite cells (because they encircle muscle fibers), start to proliferate and differentiate into myoblasts. These mononuclear cells proliferate and fuse together (primary fusion step) to create multinucleated myocytes or myotubes over the course of several days. Additional myoblasts fuse to the existing myotubes in the secondary fusion step to produce even larger myotubes which eventually align to form muscle fibers. This differentiation process is regulated by many internal and external factors. Over the course of myogenesis, expression of stem cell markers such as Pax7 is gradually lost, while the appearance of differentiation markers, such as myosin heavy chain (MyHC), muscle-regulatory factor 4 (Mrf4), and muscle creatine kinase (MCK) gradually increases. The NF-κB pathways are now known to play significant roles in this differentiation process. The canonical (or classical) NF-κB pathway is needed for myogenic cell proliferation. However, upon the loss of canonical NF-κB signaling and the activation of non-canonical (or alternative) NF-κB signaling, myoblasts stop dividing and start fusing to form multinucleated myotubes, a key event in myogenesis.

Skeletal muscle is comprised of multinucleated fibers that result from the fusion of hundreds or thousands of individual mononucleated progenitor cells. In addition to their highly specialized roles in the generation of force, individual muscle fibers are capable of extensive metabolic and functional plasticity. Skeletal muscle also exhibits robust regenerative capacity, as a means to recover from injury as well as to adapt to changing physical demands (1). A population of muscle-resident stem cells, known as satellite cells, resides within the laminin sheath encasing each muscle fiber, and is responsible for regeneration of muscle in the adult. These normally quiescent cells enter the cell cycle upon muscle injury, producing a transient and rapidly expanding population of committed progenitors or myoblasts. After several rounds of proliferation, the myoblasts enter a highly orchestrated differentiation program, wherein most exit the cell cycle, adopt biochemical and physiological characteristics of mature muscle, and fuse with each other to replace or repair the damaged tissue (Figure 1). The multiple steps in the process of muscle regeneration, beginning with satellite cell activation and ending with myoblast fusion, are all subject to separate levels of regulation, and are affected by a variety of muscle disorders and myopathies.

Investigation of the intracellular signaling pathways involved in muscle repair has been traditionally hampered by difficulties in accurately modeling the regenerative context *in vitro*. Recent developments in genetic and imaging techniques, however, have allowed new and detailed insights into many aspects of the repair process. These insights can be summarized under three major themes. First, myogenesis is comprised of several processes with distinct, and not necessarily complementary, regulatory, and signaling requirements. For instance, a pathway that promotes myoblast proliferation may have different and even inhibitory effects on the subsequent steps of myoblast differentiation, fusion, and muscle growth. Second, several cell populations co-exist with satellite cells in skeletal muscle; these other cells are either myogenic precursors [as in the case of pericytes surrounding blood vessels (2)] or non-myogenic contributors to the regenerative process, such as macrophages. Third, skeletal muscle engages in active signaling interplay with other tissue systems to maintain physiological homeostasis. Evidence in recent years has implicated the TWEAK-NF-κB signaling axis in several important functions associated with muscle damage and repair. This short review highlights both known and putative roles of TWEAK signaling in muscle biology. We emphasize recent discoveries that reflect the diverse and highly context-dependent effects of TWEAK on muscle regeneration and homeostasis. A companion article in this research topic by Sato et al. further discusses the critical importance of TWEAK signaling in skeletal muscle atrophy (3).

## Regulation of NF-κB Signaling by TWEAK

Fibroblast growth factor-inducible protein 14 (Fn14/TNFRSF12A) is classified as a member of the tumor necrosis factor receptor (TNFR) superfamily based on its ability to bind TWEAK (TNFSF12), although it bears minimal sequence homology to other TNFR superfamily members (4, 5). Fn14 is also the smallest member of the TNFR superfamily; the proteolytically processed form that is present as a transmembrane receptor has only 102 amino acids (4, 6). Furthermore, the cytoplasmic tail contains a single TNF receptor-associated factor (TRAF)-binding domain but lacks a death domain motif normally found in several other TNFR superfamily members. The adaptor proteins TRAF-1, -2, -3, and -5 are able to bind to this site, and are essential for downstream pathway activation (5, 7, 8). Given the lack of other functional domains it is likely that all TWEAK–Fn14 signal transduction is due to interaction of Fn14 with one or more of these TRAF adaptors (9). While a comprehensive screen of TNF superfamily cytokines identified TWEAK as the only ligand able to interact with Fn14 (10), a number of reports suggests that the TWEAK–Fn14 pairing is not exclusive. For example, the scavenger receptor CD163 can bind and internalize TWEAK, though there is no evidence of signal transduction activity resulting from this interaction (11). A 2003 study (12) demonstrated the ability of TWEAK to induce robust differentiation of RAW264.7 murine macrophages, which do not express Fn14 (12, 13). Other studies have demonstrated the ability of Fn14 to activate canonical NF-κB signaling in the absence of TWEAK (7, 14). Furthermore, three separate studies reported that down-regulation of Fn14 severely attenuates myoblast fusion, even in the absence of TWEAK (15–17). Nevertheless, the interaction of TWEAK with Fn14 is sufficient to activate canonical and non-canonical NF-κB pathways (9, 18, 19), so we will focus hereafter on signaling mediated by the binding of TWEAK to Fn14.

### TWEAK, the cIAP proteins, and canonical NF-κB signaling

**Figure 2:**
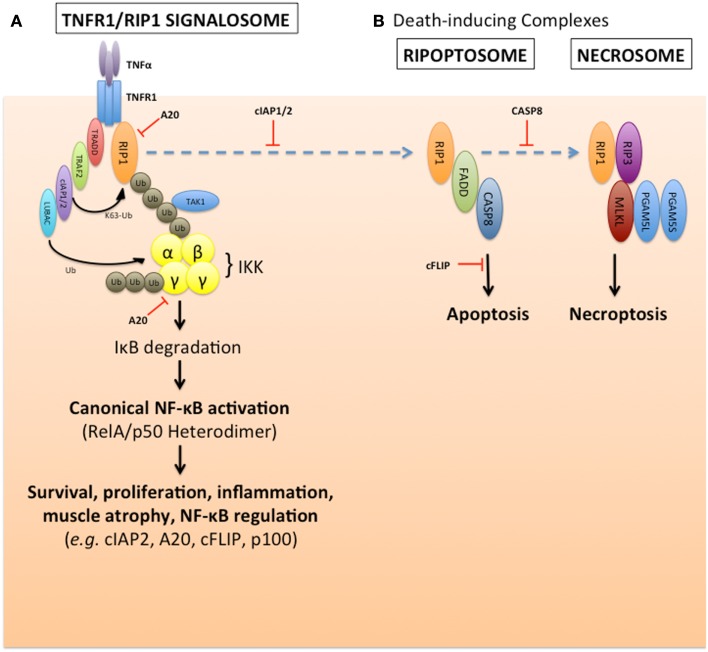
**cIAP1/2 regulation of TNFα-induced canonical NF-κB pathway activation and suppression of default death pathways**. **(A)** The E3 ubiquitin ligases cIAP1 and cIAP2 are required for TNFα activation of canonical NF-κB signaling, and to suppress TNFα-induced caspase-8 death pathway. These two cIAPs are recruited to the TNFα receptor, TNF-R1, via their association with the adaptor protein TRAF2. Upon ligand stimulation, the cIAPs promote K63-linked polyubiquitination of RIP1, which creates a signaling scaffold (or signalosome complex) to recruit the TAK1 kinase, LUBAC, otherwise known as the linear ubiquitin chain assembly complex (consisting of Sharpin, HOIL-1L, and HOIP), and the trimeric IKK kinase complex (consisting of IKKα, IKKβ, and IKKγ/NEMO). This signalosome formation results in the activation of the IKK complex, and subsequent phosphorylation and degradation of the NF-κB inhibitor, IκB, which occurs within minutes. This allows canonical NF-κB heterodimers to form and translocate into the nucleus to alter gene expression profiles over several hours, which affects many pathways such as survival, proliferation, inflammation, muscle atrophy, and NF-κB signaling itself. This pathway is subject to negative regulation by the deubiquitinase, A20, for example. **(B)** In the absence of the cIAPs, the unmodified RIP1 can form a death complex with FADD and caspase-8 known as the ripoptosome. This apoptotic death pathway can also default to a necrotic-like pathway in the absence of caspase-8, through another complex called the necrosome which involves the kinases RIP1 and RIP3, mixed lineage kinase domain-like (MLKL) and the short and long forms of the phosphatase PGAM5.

The NF-κB family consists of five transcription factor subunits, as well as a plethora of inhibitors, activators, and signal transduction molecules that function as both pathway regulators and mediators of inter-pathway cross-talk. The NF-κB subunits are RelA/p65, RelB, c-Rel, p105/p50 (NF-κB1), and p100/p52 (NF-κB2) (20). All subunits contain a Rel-homology domain near their N-termini, which confers protein dimerization and DNA-binding capabilities; however, only RelA, RelB, and c-Rel contain C-terminal transactivating domains. An NF-κB complex consists of a homodimer or heterodimer of any pair of subunits. These dimers are normally retained in the cytoplasm in an inactive state by an array of inhibitor of κB (IκB) repressor proteins. One of the better-studied NF-κB signaling axes, known as the classical or canonical pathway, principally involves signal transduction through the p50:RelA heterodimer. Upon pathway stimulation by ligands such as TNFα (Figure 2), a signaling complex forms, which leads to the activation of IκB kinase α, β, and NEMO complex (IKK). IKK catalyzes the phosphorylation and subsequent degradation of IκBα, thus allowing nuclear translocation of p50:RelA and transcriptional activation of NF-κB target genes.

Several studies over the past two decades have identified the cellular inhibitor of apoptosis 1 and 2 (cIAP1/2) proteins as regulators of multiple signal transduction pathways, including NF-κB, that are activated by TNF superfamily cytokines (19, 21–29). The inhibitors of apoptosis (IAP) were identified based on their homology to viral IAPs (30–32), and contain one or more N-terminal baculoviral IAP repeat (BIR) homology domains. In addition, cIAP1/2 were independently identified based on their association with the TNF receptor 2 (TNF-R2) and the adaptors TRAF1 and TRAF2 (21). These two IAPs are recruited to the various TNF superfamily receptors through direct interaction with TRAF1, TRAF2, or TRAF6 (19, 33–36). The cIAP1/2 proteins contain single C-terminal RING E3 ubiquitin ligase moieties (37), as well as ubiquitin-binding UBA domains (38), whose functions have been the focus of considerable research interest. In most cells, cIAP1 triggers the constitutive lysine-48 (K48)-ubiquitination and degradation of cIAP2 (39); thus, cIAP2 expression is low in most non-lymphoid tissues unless cIAP1 is absent, or unless cIAP2 expression is induced (40–42). Nevertheless, in many scenarios cIAP1 and cIAP2 function redundantly (28). In the context of NF-κB signaling induced by a prototypical ligand such as TNFα, the TNF receptor 1 (TNF-R1) recruits a signal transduction complex consisting of TNF-R1-associated DEATH domain (TRADD), the kinase/scaffold protein receptor-interacting protein 1 (RIP1), and TRAF2. TRAF2 recruits cIAP1/2, which in turn catalyze the polyubiquitination of RIP1 by K63- and K11-mediated linkages (43). The K63- and K11-linked chains on RIP1 serve as scaffolds for the assembly of an IKK complex consisting of TAB1, TAK1, NEMO, and IKKα,β. This complex phosphorylates IκBα, thus completing the signal transduction process upstream of the NF-κB transcription factors (Figure 2). When cIAP1/2 are depleted, either by genetic or pharmacological means (such as through the use of IAP antagonists known as SMAC mimetic compounds or SMCs), RIP1 instead activates a pro-apoptotic complex, referred to as the ripoptosome. This death complex consists of de-ubiquitinated RIP1, the DEATH domain-containing adaptor protein FADD, and caspase-8 (44, 45) (Figure 2).

The receptor Fn14 is an effector of signaling through its ability to recruit TRAF-1, -2, -3, and -5. At sufficiently high concentrations, soluble TWEAK triggers IκB phosphorylation and degradation, as well as p65 phosphorylation and nuclear translocation, events that are typical of the canonical NF-κB pathway activation (7). As Fn14 lacks a death domain, it is unable to directly recruit a death-signaling complex. Instead, apoptosis results from the NF-κB-stimulated release of TNFα, which induces apoptosis in a manner requiring RIP1 and FADD (46). Cancer cells that have been “primed” with TWEAK are sensitized to TNFα-induced cell death owing to the depletion of cIAP1 and TRAF2 proteins (18). Evidence is accumulating that canonical NF-κB activation and cell death are consequences of pathological, rather than physiological, levels of TWEAK or Fn14. At low (physiological) concentrations, TWEAK is unable to activate canonical NF-κB, but still produces robust activation of the non-canonical pathway (17, 47), as described in the next section. Remarkably however, membrane-bound TWEAK is highly capable of activating canonical NF-κB signaling (47), suggesting that juxtacrine signaling may produce considerably different effects on target cells. Furthermore, Fc-TWEAK or Fc-Fn14 fusion constructs, which have a high propensity to multimerize, are able to activate Fn14 and cause significant canonical NF-κB activation with pathological outcomes (47–49). These negative consequences of TWEAK signaling are also seen upon Fn14 upregulation due to stress or injury, even when TWEAK levels remain unchanged. This is due to the greater chance for receptor oligomerization and clustering to occur, which is needed to induce downstream signaling events (50).

During myogenesis, canonical NF-κB activity promotes myoblast proliferation and inhibits differentiation [reviewed elsewhere (51–53), and see Figures 1 and 4 for illustration]. These effects are important during the early phases of muscle regeneration, where efficient repair necessitates rapid expansion of the myoblast population. Notably, following muscle damage, the first wave of inflammatory cells release a plethora of inflammatory cytokines, such as TNFα, IL-6, and TWEAK, which are potent activators of NF-κB signaling (54, 55). During chronic regenerative cycles observed in certain muscle disorders, such as Duchenne muscular dystrophy, the continued presence of such inflammatory cytokines both impairs muscle repair and aggravates the resulting pathology (56). This differential effect of transient and chronic cytokine signaling will be discussed later in this review, in the context of non-myogenic contributors to myogenesis.

### TWEAK and non-canonical NF-κB signaling

**Figure 3:**
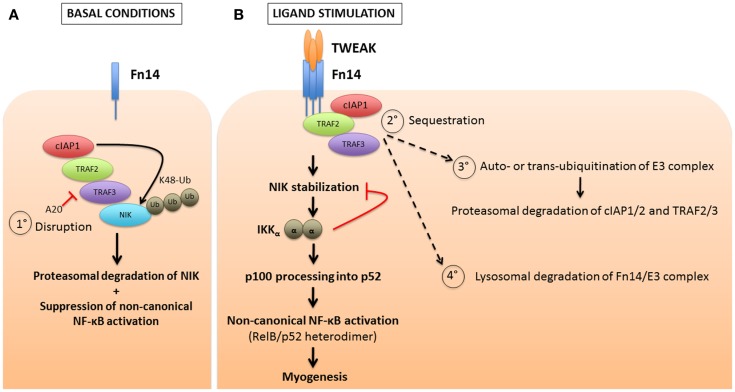
**cIAP1/2 regulation of TWEAK-induced non-canonical NF-κB pathway activation**. **(A)** Contrary to the canonical NF-κB pathway for which cIAP1/2 are positive regulators, these two E3 ubiquitin ligases act, via the bridging molecules TRAF2 and TRAF3, as negative regulators of the non-canonical NF-κB by continuously degrading the NF-κB-inducing kinase, NIK. This occurs through the attachment of K48-linked polyubiquitin chains and the targeting of NIK to the proteasome, under basal or non-stimulated conditions. One mechanism (process 1**°**) to reverse this inhibitory effect is through A20 mediated disruption of the cIAP-TRAF complex, which would presumably lead to ligand-independent activation of the non-canonical NF-κB pathway. **(B)** In most instances, upon stimulation of a TNF receptor superfamily member by its ligand, the cIAPs and TRAFs are recruited away from the cytosolic reactions and sequestered at the plasma membrane (process 2**°**). This allows for the stabilization of NIK, the formation of IKKα homodimers, and ultimately the partial processing of p100 into p52. RelB and p52 then dimerize to form an active, functional NF-κB transcription factor complex. Several models of receptor-mediated non-canonical NF-κB activation have been proposed, which include the cIAPs inducing K48-linked ubiquitination of themselves and the TRAFs, resulting in their proteasomal degradation (process 3**°**). Alternatively, the receptor-mediated endocytosis of the TWEAK-Fn14 complex results in lysosomal degradation of the cIAPs and TRAFs (process 4**°**). This loss of cIAP and TRAF adaptors may impact other pathways, such as CD40L signaling through CD40, that also require these adaptors.

Transcriptional activity in the non-canonical NF-κB pathway is mediated by the p52:RelB heterodimer. This signaling axis is tightly regulated by the controlled processing of p100 into p52. Under basal conditions, a ubiquitin ligase complex consisting of TRAF2, TRAF3, and cIAP1/2 catalyzes the constitutive K48-ubiquitination and consequent degradation of the NF-κB inducing kinase (NIK), which is essential for activation of the non-canonical pathway (33, 57). Upon non-canonical NF-κB stimulation by a variety of ligands [including TWEAK, BAFF, CD40 ligand (CD40L), and RANKL], TRAF2, TRAF3, and cIAP1/2 are sequestered to the corresponding membrane-bound TNF superfamily receptor. Here, K48-ubiquitination of TRAF3 by cIAP1/2 leads to auto-inactivation of the complex and stabilization of cytosolic NIK (58). NIK in turn phosphorylates IKKα, which activates p100 and leads to its partial proteasomal processing to p52. The p52:RelB dimer is then released for nuclear translocation and gene transactivation (Figure 3). TWEAK signaling subsequently triggers the degradation of TRAF2, TRAF3, and cIAP1/2 through both proteasomal and lysosomal pathways (17, 18, 59, 60). The lysosomal degradation mechanism may represent a separate mode of NF-κB activation unique to TWEAK, since inhibiting lysosomal protein degradation is sufficient to completely prevent p100 processing (18). Unlike the stimulation of the canonical pathway, which is quite rapid, the non-canonical pathway is gradually activated over several hours, possibly due to the requirement for *de novo* NIK translation and accumulation. The lysosomal degradation of cIAP1 and TRAF2 by TWEAK impairs NF-κB activation by other cytokines that require these adaptors; thus, TWEAK sensitizes cancer cells to TNFα-induced apoptosis through activation of caspase-8 (8, 18, 61). The cIAPs are thus considered to be negative regulators of the non-canonical NF-κB pathway, through their constitutive effects on NIK degradation. The binding of TWEAK to Fn14 then relieves this cIAP1/2 suppression by recruiting the TRAFs and cIAPs to the receptor, away from NIK. This membrane receptor sequestration of the cIAPs and TRAFs may be sufficient for NIK stabilization, or may require further degradation and loss of those factors to fully activate NIK as illustrated in Figure 3.

## TWEAK and cIAP1 as Regulators of Myoblast Fusion

While the functions of canonical NF-κB signaling in muscle regeneration and atrophy have been investigated extensively over the years (52, 62, 63), very few studies have examined the role of non-canonical NF-κB in skeletal muscle. In 2001, a paper (64) suggested that NIK and IKKα promote differentiation of the rat L6E9 myoblast cell line. More recently, the non-canonical NF-κB signaling was implicated in muscle resistance to metabolic stress (65), and as a factor specifying the oxidative mode of glucose metabolism in muscle fibers (66). We had observed that primary myoblasts from cIAP1^−^*^/^*^−^mice [note that skeletal muscle does not express cIAP2 (28)] exhibit constitutively elevated canonical and non-canonical NF-κB activity. We reasoned that upon differentiation of cIAP1^−^*^/^*^−^myoblasts into myotubes, which are the *in vitro* analogs of muscle fibers, both canonical and non-canonical NF-κB pathways should produce separate respective phenotypes.

**Figure 4:**
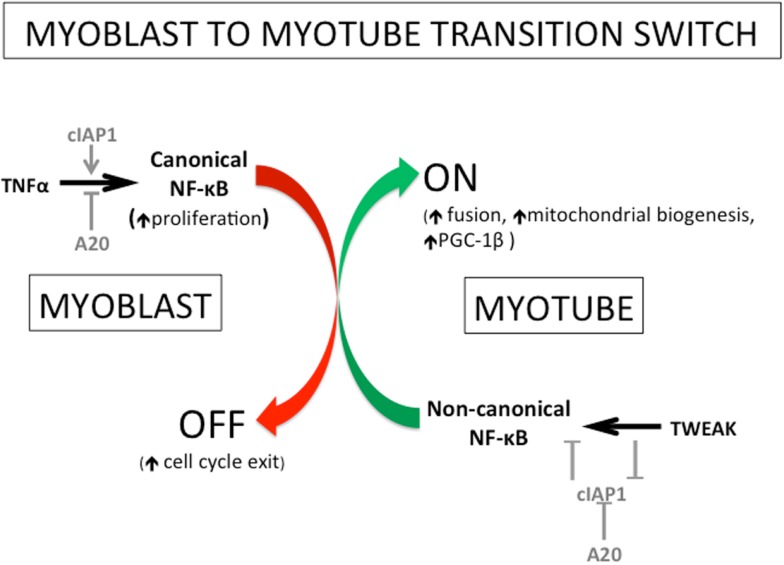
**Roles of TNFα, TWEAK, cIAP1/2, and A20, in myogenesis in the regulation of switching from canonical to non-canonical NF-κB signaling**. The transition from myoblast to myotube has been known for several years to involve a switch from canonical to non-canonical NF-κB signaling. More recently, several of the key players and the mechanisms of action for this switch have been identified. The promotion of TNFα-mediated canonical NF-κB signaling involves the positive regulation by the E3 ubiquitin ligase cIAP1/2 and the negative regulation by the deubiquitinase A20. The non-canonical pathway is oppositely controlled: it is negatively regulated by cIAP1/2 and positively regulated by A20. Interestingly, the cytokine TWEAK (a pathological factor in muscle atrophy) has been recently shown to stimulate non-canonical NF-κB signaling by removing the negative regulation of cIAP1/2 on that NF-κB pathway. Physiological levels of TWEAK, in fact, promote myogenesis through the enhancement of myoblast fusion amongst other things. The non-canonical NF-κB pathway has been shown previously to regulate mitochondrial biogenesis and to promote myotube maintenance, and recently shown to do so through the NF-κB-mediated induction of the mitochondrial regulator PPAR-γ co-activator 1β (PGC-1β). Figure adapted from Ref. (65).

The initial outcome of our experiments was unexpected. While there was a clear delay in cell cycle exit and differentiation of cIAP1^−^*^/^*^−^myoblasts, the resulting myotubes were characterized by significant hypernucleation and increased myotube size, indicative of a robust fusion response. Subsequent experiments showed that the elevated non-canonical NF-κB activity, resulting from the loss of cIAP1, was responsible for this effect (17). This observation highlighted a disparity between immortalized myoblast cell lines (C2C12) and primary myoblasts. In C2C12 cells, p100 processing to p52 increases over the time course of differentiation (65). In contrast, primary cells exhibit the greatest p100 processing at the myoblast stage; processing diminishes markedly as muscle fibers form (17). The increased myoblast fusion observed in cIAP1^−^*^/^*^−^cells could be recapitulated using low doses of TWEAK. At low concentrations, exogenous TWEAK led to robust activation of the non-canonical NF-κB pathway. At higher concentrations, TWEAK activated both canonical and non-canonical pathways. The requirement for high TWEAK concentrations to activate canonical NF-κB suggests that this pathway represents a secondary mode of signaling for TWEAK. In order to further investigate the physiological consequences or TWEAK activity *in vivo*, we employed the snake venom cardiotoxin (CTX) model of muscle injury, which involves the direct injection of CTX into muscle. This treatment causes rapid development of focal necrotic lesions, but also initiates a robust regenerative response. Following CTX injection, the cIAP1^−^*^/^*^−^muscle exhibits a slight increase in average muscle fiber size as compared to wildtype controls, but not to the same robust extent observed *in vitro* (17). This is likely an outcome of the interplay and functional antagonism between both NF-κB pathways. TWEAK administered by micro-osmotic pump produced greater increases in fiber size than did the loss of cIAP1, further indicating the preference of TWEAK for non-canonical NF-κB signaling. In regenerating muscle, the window of regenerative opportunity is very narrow; the majority of myogenesis occurs within 4 days of the injury. A delay in the development of fusion competence – as can be caused by elevated canonical NF-κB activity – may be sufficient to reduce the muscle’s regenerative potential. Collectively, the data indicate that enhanced myogenesis is best achieved by attenuating the canonical NF-κB pathway, and promoting fusion through the non-canonical corollary [(17); see also Figure 4].

The evidence to date shows that both canonical and non-canonical NF-κB pathways are concurrently active in proliferating and differentiating mouse myoblasts. During the process of muscle differentiation, both pathways are similarly inactivated. However, these NF-κB pathways clearly have complementary but opposing functions in muscle regeneration (Figures 1 and 4). While the canonical pathway is important during myoblast proliferation (67), the reported effects of non-canonical NF-κB signaling on stress resistance (65), metabolism (66), and fusion (17) are all features specific to developing muscle fibers, rather than to myoblasts. Therefore, a model can be proposed in which canonical NF-κB activity is switched off to suppress myoblast proliferation thereby allowing for their differentiation. At this point, non-canonical NF-κB predominates, likely driven by TWEAK stimulation, to promote the formation of myotubes, while also aiding in their maintenance. The canonical NF-κB pathway may in fact prime the non-canonical NF-κB pathway by inducing the expression of p100, the precursor to p52. It is thus possible that p52:RelB (non-canonical NF-κB) activity then serves to further push myoblast fusion, by inducing expression of transcriptional targets whose functions are only observed in later stages of the differentiation process. With the exception of PGC-1β (66), the relevant transcriptional targets of non-canonical NF-κB in this context, and the effectors of TWEAK-driven myoblast fusion, are unknown. Another possibility is that, though p100/52 expression is high in myoblasts, activity of this pathway may be inhibited in myoblasts, and subsequently de-repressed during differentiation. A potential mechanism for the switch between canonical and non-canonical pathways involves the deubiquitinase A20. The A20 protein inhibits canonical NF-κB signaling by removing the K63-ubiquitin chains on RIP1 that are essential for its function as an adaptor (68). Conversely, A20 disrupts the cIAP1/2–TRAF2/3 ubiquitin ligase complex, thus preventing NIK degradation and promoting non-canonical NF-κB signaling (69). Since A20 has been shown to be upregulated during muscle cell differentiation (70), it is possible that A20 is essential for normal regeneration.

Knockout studies have revealed disparities between the muscle-intrinsic effects of TWEAK and Fn14 on regeneration. Upon muscle injury, TWEAK^−^*^/^*^−^mice exhibit more rapid muscle regeneration than wildtype controls; in contrast, mice over-expressing TWEAK under a muscle-specific promoter exhibit slower regeneration (71). However, TWEAK is not normally expressed in myoblasts either *in vivo* or *in vitro* (15, 17), suggesting that the phenotype observed in the knockout mice may result from decreased paracrine signaling from other TWEAK-producing cells such as macrophages. Fn14 expression in muscle is clearly induced during regeneration and atrophy (16, 50), and mice lacking Fn14 exhibit considerably impaired regeneration. This effect can be recapitulated *in vitro* in the absence of TWEAK (16, 17), highlighting the possibility that Fn14 may play essential roles independent of TWEAK.

**Figure 5:**
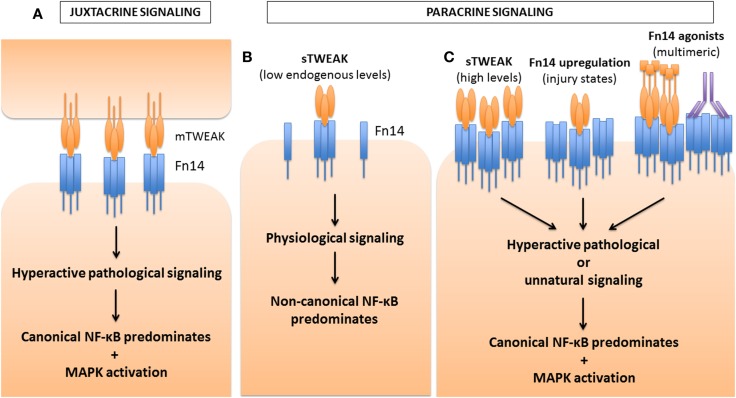
**Differential activation of NF-κB pathways by TWEAK based on strength of Fn14 signaling**. TWEAK can signal either in a juxtacrine manner, as a membrane-bound ligand, mTWEAK **(A)**, or in a paracrine manner as a soluble ligand, sTWEAK **(B,C)**. Depending on the concentration of ligand or receptor, or the propensity of the Fn14 ligand to oligomerize the receptor and form signaling clusters, differential activation of the NF-κB pathways occurs. **(A)** During juxtacrine signaling, mTWEAK favors the clustering of Fn14 on the opposite cell, leading to hyperactive signaling for which canonical NF-κB signaling predominates over non-canonical signaling. MAPK activation also occurs. **(B)** Under physiological conditions, low endogenous levels of sTWEAK signal in a paracrine manner, leading predominantly to non-canonical NF-κB activation. **(C)** However, under certain pathological or experimental conditions, sTWEAK can also lead to hyperactive signaling leading to canonical NF-κB and MAPK signaling. For example, high concentrations of TWEAK, applied to myoblasts or myotubes *ex vivo*, hinder differentiation and cause atrophy, respectively. Transgenic over-expression of TWEAK in mice also leads to pathological consequences. In a variety of injury states (such as denervation-induced muscle atrophy), the receptor Fn14 is induced, which then is followed by and pathological responses to endogenous levels of TWEAK. Furthermore, recombinant TWEAK fusion proteins with the ability to multimerize, or Fn14 agonistic antibodies, can both promote receptor clustering and the activation of the canonical NF-κB pathway. The pathways leading to pathological activation of the canonical NF-κB pathway are still poorly defined, but are thought to arise either from pathway cross-talk, such as that seen with NIK or IKK activation of the canonical mediators, or through the adaptor and E3 ubiquitin ligase TRAF6. In addition, activation of MAPK pathways may also contribute to the pathology observed.

The application of TWEAK/Fn14 therapeutics in the context of muscle regeneration may at first appear somewhat counterintuitive, until the specific mechanistic intent is examined closely, and hypotheses are tested. Most of the published reports on TWEAK highlight its aggravating role in muscle (72, 73), liver (74), kidney (75) and neurological (76) regeneration or repair. A consistent feature of these studies however, is the pathologically elevated levels of TWEAK signaling, due either to the experimental intervention (transgenic over-expression of TWEAK) or a chronic, localized over-production of cytokine (77). However, we propose that transient TWEAK/Fn14 activation at physiological levels may prove beneficial (Figure 5). Consistent with this idea, a number of studies have shown that TWEAK can act as a mitogen to stimulate proliferation of progenitor cells (15, 78–81). In particular, soluble TWEAK, particularly at low concentrations, preferentially activates the non-canonical NF-κB pathway, whereas high concentrations are sufficient to mobilize both canonical and non-canonical pathways (17, 48). Therapeutic activation of the non-canonical pathway has been suggested in a number of separate contexts. For example, TWEAK promotes lymphocyte and T cell recruitment to the kidney following renal injury by inducing expression of the chemokine CCL21 in a non-canonical NF-κB-specific manner (82, 83). TWEAK is also important to prevent certain lymphoproliferative disorders that lead to impaired antibody responses (84, 85). A better understanding of the mechanisms of TWEAK signaling should permit an informed tailoring of its uses, such as with agonistic and antagonistic antibodies (48), for particular therapeutic applications.

## NF-κB, NFAT Signaling, and the Promotion of Myoblast Fusion

Extracellular calcium is one of the earliest known regulators of myoblast fusion. In 1969, Shainberg and colleagues (86) demonstrated that fusion of chick myoblasts could be reversibly blocked by removing Ca^2+^ ions from the growth medium. These results were confirmed in multiple species (87–89). Importantly, it was shown that extracellular calcium is not required for myoblast cell cycle exit or for muscle-specific gene expression (90–93). Calcium was subsequently identified as a potent activator of the nuclear factor of activated T cells (NFAT) transcription factor pathway. Out of five proteins in this family, three – NFAT-c1, -c2, and -c3 – are regulated by calcium and expressed in skeletal muscle (93). Ca^2+^-activation of the phosphatase calcineurin leads to the dephosphorylation of NFAT proteins. These then translocate to the nucleus where they activate the transcription of a range of NFAT target genes (94). Multiple stimuli that potentiate the Ca^2+^-calcineurin-NFAT signaling axis, such as calcium ionophores (95), increase myoblast fusion; conversely, substances that deplete intracellular or extracellular calcium stores, such as EDTA and thapsigargin, impair myoblast fusion (96, 97). A notable aspect of NFAT transcription factors is that they mediate different aspects of myoblast fusion. Whereas NFATc3 is calcium-responsive in myoblasts, NFATc1 and NFATc2 are active in nascent myotubes (93). This suggests that they differentially regulate primary fusion (myoblast–myoblast) and secondary (myoblast–myotube) fusion events (Figure 1). Consistent with this hypothesis, the protein four-and-a-half LIM 1 (FHL1) is a co-factor of NFATc1; over-expression of FHL1 increases myoblast fusion *in vitro* and *in vivo* (98). Muscle fiber formation is also impaired in *NFATc2*^−^*^/^*^−^mice (99). Collectively the data indicate a hierarchical process of fusion, whereby primary myotubes, formed under control of NFATc3, recruit further myoblasts in an NFATc1- or NFATc3-specific manner for formation of secondary myotubes and continued myogenesis.

Evidence from some published reports suggest that the TWEAK/Fn14/cIAP signaling axis may act through one or more NFAT pathways to regulate myoblast fusion. Upon inactivation of the non-canonical NF-κB pathway in wildtype myoblasts, myotubes still form, but are small and have reduced numbers of nuclei (17). This phenotype is similar to that observed with *NFATc2*^−^*^/^*^−^cells (99), where myogenesis stalls at the primary myotube phase. The existence of cross-talk between the structurally similar NFAT and NF-κB transcription factors is well-established (100–102). A recent study indicated that RANKL, which like TWEAK activates both canonical and non-canonical NF-κB pathways in osteoclasts, induces expression of NFATc1 in a manner that requires NIK (103), suggesting that NFATc1 may be a target of the non-canonical pathway. Furthermore, cIAP1/2 was shown to suppress NFATc1 expression in response to RANKL; conversely, loss of cIAP1/2 led to upregulation of NFATc1in osteoclasts (104).

Our understanding of molecular triggers and signaling pathways that are critical to myoblast fusion is still very limited. While the list of known effectors of fusion is extensive [as has been categorized in other recent reviews (105, 106)], a coherent picture of timing, mechanism, and relative importance has yet to emerge. It seems likely that the TWEAK-NF-κB signaling axis converges with the transcriptional upregulation of one or more muscle-derived cytokines or chemokines (referred to as “myokines”). A search for known targets of the non-canonical NF-κB pathway, including such molecules as CCL19 and CCL21 (83), should provide further insight into the placement of NF-κB within the stepwise processes of fusion.

## Innate Immunity and Muscle Regeneration Mediated by the Cytokines IL-4, IL-10, and IL-13

In the context of regenerating muscle, a number of recent papers have examined the interplay between muscle cells and multiple non-muscle lineages that participate in the regenerative process. Following muscle injury, an inflammatory response emerges, which involves the infiltration of myeloid cell types such as eosinophils, basophils, mast cells, macrophages, and leukocytes (107, 108). These cells release a medley of cytokines and chemokines; the leukocytes in particular are robust sources of TWEAK (109, 110). Broadly speaking, the damage-associated innate response is structured such that the early infiltrating cells, dominated by CD68-expressing “M1” macrophages, produce pro-inflammatory cytokines such as IL-6, TNFα, and IL-1β (54). The timing of M1 influx correlates with the activation of satellite cells and proliferation of myoblasts, a process that is enhanced by canonical NF-κB activation. The M1 macrophages are subsequently replaced with CD163-expressing “M2” macrophages, which promote muscle growth through the secretion of IL-4, IL-10, and IL-13. The M2 response occurs during the phase of muscle regeneration predominated by myoblast fusion, which may be enhanced by M2-derived cytokines such as IL-4 and IL-13.

The various contributions of inflammatory and lymphoid cells to the course of muscle regeneration have been assessed in injury models. A commonly used animal model of muscle injury is the *mdx* mouse, which exhibits many of the hallmark symptoms and pathology of the human disorder known as Duchenne muscular dystrophy. In mice and humans, the disease results from loss of the structural protein dystrophin, which leads to increased muscle fragility and continued cycles of injury and regeneration (111). This creates a chronic inflammatory milieu in muscle, which both aggravates and perpetuates the pathology. In *mdx* mice depleted of macrophages during the early stages of the disease, muscle injury is significantly reduced (112). Similar outcomes are observed in *mdx* mice depleted of CD8-positive cytotoxic T cells alone (113), or of both CD4-positive helper and CD8-positive T cell populations (114). An important point of note, however, is that these studies describe the outcome of short-term depletion of these cell populations on the dystrophic phenotype. In an analysis of *mdx* mice crossed with *scid* mice (lacking both mature T and B cells), no differences in muscle fiber size, percentage of regenerated fibers, or muscle force were observed as compared to immunocompetent *mdx* mice (115). In contrast, mice depleted of monocytes and macrophages, using a targeted cytotoxic diphtheria toxin approach, exhibit severely impaired muscle regeneration (116). These observations are consistent with the biphasic and important roles of M1 and M2 macrophages in the regenerative process.

Also relevant to this stage of regeneration is a recently identified population of fibro/adipogenic progenitors (FAPs), which are essential contributors to normal muscle regeneration following acute trauma (117–122). These FAPs, like satellite cells, are activated following muscle injury, and proliferate in response to IL-4 and IL-13 secreted by eosinophils (123). IL-4 also specifies the fate of FAPs as phagocytes rather than fat-generating adipocytes. In the absence of IL-4/IL-13-secreting eosinophils or in an IL-4 receptor alpha-knockout (*IL-4Rα*^−^*^/^*^−^) background, muscle regeneration is severely impaired, at least in part due to excessive deposition of FAP-generated brown fat. There are currently no studies explicitly examining the relationship between TWEAK and FAPs during myogenesis; nevertheless corollary evidence from other tissue systems suggests that TWEAK may promote muscle regeneration through regulation of FAP differentiation. TWEAK and Fn14 are expressed in adipocytes (124, 125), and TWEAK inhibits adipocyte differentiation (126). This occurs at least in part through the blunting of pro-inflammatory and pro-adipogenic signaling induced through the canonical NF-κB pathway by TNFα (125–127). Still further, TWEAK synergizes with IL-13 as a fibroblast mitogen (127, 128). Given the overlap between the influx of TWEAK-expressing M2 macrophages and FAP activation during regeneration, it seems likely that the pro-regenerative context established by both cell types may involve low levels of secreted TWEAK as a paracrine regulator of muscle regeneration.

Given the upregulation of Fn14 following muscle injury, and the influx of TWEAK-expressing myeloid cells, it is likely that the non-canonical NF-κB signaling has a direct influence on the course of muscle repair. The highly orchestrated nature of the innate immune response in damaged muscle, which occurs in synchrony with the course of muscle differentiation and fusion, is critical to normal muscle regeneration. This timing is controlled by IL-10 (129), which deactivates M1 macrophages; and by AMPKα1, which is required for macrophage acquisition of an M2 phenotype (130). Given that such timing is disrupted in chronic degenerative muscle diseases and myopathies, factors that skew the population distribution in favor of an M2 phenotype may improve muscle regeneration in disease conditions. This hypothesis is supported by certain acute experiments in which administration of exogenous IL-4 (123) or IL-10 (131) was found to promote necrotic cell clearance and muscle regrowth.

Recently we evaluated the effect of cIAP1 loss on muscle function in the *mdx* mouse model of Duchenne muscular dystrophy (132). In *cIAP1*^−^*^/^*^−^*mdx* double-mutant mice, muscle degeneration was attenuated in some muscle groups, particularly the soleus and diaphragm. The outcome was that double-mutant mice exhibited improved muscle resiliency and exercise endurance as compared to the *mdx* controls. These results were accompanied by a reduction in pro-inflammatory M1 macrophages, and an increase in pro-regenerative M2 macrophages in muscle tissue. These results suggest that non-canonical NF-κB activation, through loss of cIAP1 can mediate diverse effects that converge to improve muscle regeneration and function. It remains to be seen if low doses of TWEAK, which would more specifically target the non-canonical pathway, can recapitulate these positive effects on muscle regeneration.

## TWEAK Signaling in Diabetes and Muscle Regeneration

Skeletal muscle is responsible for the uptake of 80% of blood glucose (133–135); consequently, the outcome of prolonged insulin resistance in muscle is primarily type 2 diabetes (136). The presence of excessive fat deposits is associated with both onset and progression of type 2 diabetes (137). It was recently discovered that high-glucose diets trigger the differentiation of multipotent myoblasts into adipocytes (138). While the resulting fat deposits can accelerate insulin resistance through autocrine release of TNFα, TWEAK can inhibit this process by blocking TNF-mediated activation of JNK (139). TWEAK is constitutively expressed in adipose tissue (124, 125), suggesting that it actively antagonizes the process of insulin resistance. Evidence for this is shown by a recent study demonstrating that reduced levels of TWEAK correlate with increased risk of diabetes (140), at least in part by reducing autocrine release of TNFα from adipocytes (141). Overall, these findings suggest that TWEAK can operate in a feed-forward mechanism to both promote muscle regeneration and attenuate the pathogenesis of diabetes.

While TWEAK-expressing adipocytes may be beneficial for the purposes of insulin tolerance, it is naturally preferable to reduce fat deposition in muscle altogether. This is consistent with a beneficial role for FAPs in muscle regeneration when the differentiation choice toward adipocytes is blocked (123, 142, 143). Remarkably, TWEAK is a potent inhibitor of adipocyte differentiation and functions, unlike TNFα, without affecting glucose uptake or cytokine release (126). Collectively, the data show that TWEAK signaling can positively regulate homeostasis by improving glucose tolerance in muscle, reducing fat deposition, and reducing adipocyte differentiation of FAP cells during muscle regeneration.

## Conclusion

The array of recent discoveries on the functions and mechanisms of action of TWEAK offer several intriguing possibilities into both the frontiers of new biology and the potential for therapeutic interventions. The ability of TWEAK/Fn14 to preferentially activate the non-canonical over the canonical NF-κB pathway (17) places TWEAK in a category of TNF superfamily members along with BAFF, CD40L, RANKL, and lymphotoxin β (144). This implicates TWEAK in immunological functions that have, to date, been explored only briefly. The preferential activation of the canonical pathway by membrane-bound TWEAK and the upregulation of Fn14 upon injury are likely the causes of most TWEAK-associated pathology (47, 50). Thus, the paradox of TWEAK as a beneficial and deleterious cytokine becomes a matter of degree: whereas low concentrations of soluble TWEAK can be beneficial for immunological and regenerative purposes, high levels of TWEAK or Fn14 may have pathological consequences, and require intervention using neutralizing antibodies or TWEAK inhibitors.

## Conflict of Interest Statement

The authors declare that the research was conducted in the absence of any commercial or financial relationships that could be construed as a potential conflict of interest.
